# An Unusual Case of Bilateral Empyema Associated with Bee Sting

**DOI:** 10.1155/2014/985720

**Published:** 2014-02-16

**Authors:** Srinivasa Kaligonahalli Venkataramanappa, Amban Gowda, Srinivas Raju, Vijeth Harihar

**Affiliations:** Department of General Medicine, Dr.B.R. Ambedkar Medical College, Bangalore 560045, India

## Abstract

Bee sting in most situations is life threatening. Spectrum of bee sting ranges from mild local reaction to death. The literature regarding the bee sting disease from India is sparse. The rare manifestations of the disease include encephalitis, polyneuritis, myocardial infarction, pulmonary edema, bleeding manifestations, and renal failure. Bee sting infections are rare and no field studies have been performed to determine the exact sequence of events that lead to infection of bee stings and if not treated properly can lead to fatal outcomes. Here we present a case of unusual bilateral empyema associated with bee sting.

## 1. Introduction

The severity and duration of a bee sting reaction can vary from one person to another and at different occurrences in the same individual. These reactions were encountered in only 5% of the patients [1]. The spectrum of bee sting disease ranges from mild local reaction to death. The literature regarding the bee sting disease from India is sparse. Bee venom has many different effects on the human body. This is based on the dose of the bee venom. Bee venom, in certain cases, can have very strong toxic effects on humans. For a person who is hypersensitive to bee venom, even one sting can cause a serious or fatal reaction. Here we are reporting a case of bilateral empyema associated with bee sting.

## 2. Case Report

**Figure 1:**
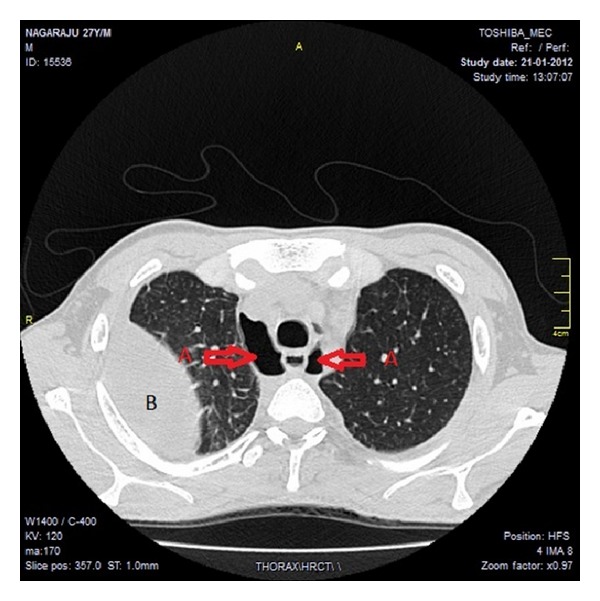
HRCT thorax (a) A indicating air in the mediastinum, (b) B indicating collection of pus in the pleural cavity.

**Figure 2:**
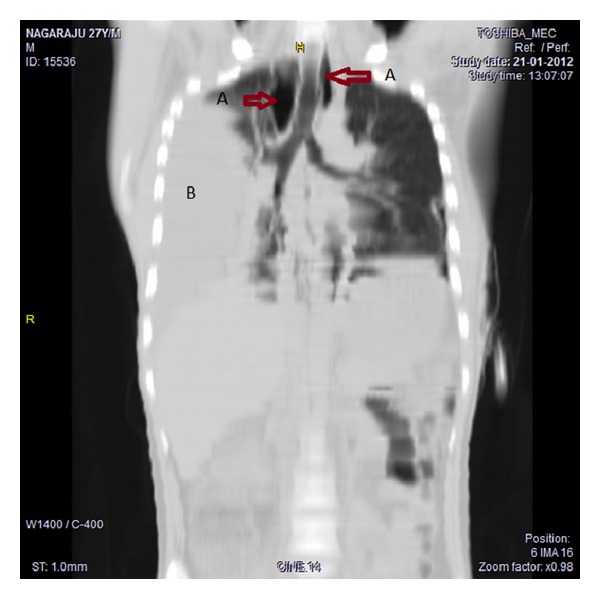
HRCT thorax (a) A indicating air in the mediastinum, (b) B indicating collection of pus in the pleural cavity.

A 27-year-old male presented with high grade fever, dyspnea, and dysphagia of three-day duration. On examination pulse was 110 beats/min, BP 130/90 mm of Hg, oxygen saturation was 96%; decreased breath sounds were heard in the left lower lung field areas. On the day of admission he developed pain abdomen and worsening of dyspnoea, oxygen saturation reduced to 86%, breath sounds were decreased in the left basal areas, and tenderness was present in left hypochondrium. Chest X-ray was suggestive of left hydropneumothorax and HRCT thorax revealed mediastinitis with left sided empyema (Figures 1 and 2); intercostal drainage tube was inserted, which revealed frank pus. After three days of antibiotic therapy fever, dyspnoea and dysphagia were still persistent, repeat chest X-ray revealed right sided pyothorax, and ICD was inserted on right side. Patient was on empirical antibiotic therapy; patient developed fever and breathlessness. Blood and empyema Culture results were negative. On reevaluation of the patient, he revealed the history of bee sting in the oral cavity three days prior to admission, ENT examination showed oedematous arytenoids leading to mediastinitis and bilateral empyema, barium swallow was performed which was normal, and oesophageal perforation was ruled out. Patient was treated with piperacillin plus tazobactam, metronidazole, and intravenous steroids for 2 weeks. Patient started improving within 48 hours of therapy and was afebrile after 5 days with complete clinical and radiological resolution by the 10th day of therapy. Patient was discharged on the 14th day and steroids were tapered over the same period. Patient is asymptomatic since then. Laboratory investigations revealed haemoglobin of 13.7 gm%, white blood cell (WBC) count of 11,200/mm^3^, differential count of 87% neutrophils, platelet count of 2,01,000/mm^3^, an erythrocyte sedimentation rate (ESR) of 60 mm in 1 hour. Liver function test revealed total bilirubin 1.9 mg/dL, direct 0.8 mg/dL, SGOT-49 IU/L, SGPT-54 IU/L, alkaline phosphatase 80 U/L; serum amylase 28 U/L, serum lipase-65 U/L; B urea 68 mg/dL, S creatinine 1 mg/dL. HIV test was nonreactive. Pleural fluid analysis revealed frank pus, sugar 15 mg/dL, proteins 2.3 gms/dL, cell count 2000 cells/cumm, plenty of neutrophils with occasional macrophages, ADA-26.1 U/L. Pleural fluid cultures yielded no fungi or bacterial pathogens. Pleural fluid triglycerides levels were 32 mg/dL.

## 3. Discussion

The venom of winged Hymenoptera contains over 30 individual compounds. These include biogenic amines (acetylcholine, dopamine, histamine, norepinephrine, and serotonin), polypeptides or protein toxins (apamin, melittin, and kinins), and enzymes (hyaluronidase and phospholipases). Though widely studied in western and African countries regarding various allergic reactions of bee sting, the literature from India is sparse and most of the cases were single case reports. Two kinds of reactions are usually associated with bee stings and those are either local or systemic. A local reaction is generally characterized by pain, swelling, redness, itching, and a wheal surrounding the wound made by the sting; principally it is of type 1 anaphylactic reaction mediated by mast cells. Serum sickness type of reaction is more likely after an episode of multiple stings (malaise, fever, joint pains, skin rashes, swelling of lymph glands, and kidney injury) and may develop three to ten days after a sting [2]. Adults whose reactions include urticaria, obstruction of the upper or lower airway, or hypotension and children whose reactions include obstruction of the upper or lower airway or hypotension have an increased risk of future systemic reactions to stings [3]. Occurrence of life threatening wheezing, laryngeal edema, or anaphylactic shock in stings, produced by a substance called precipitin. They are considered extremely rare [4]. Bee sting infections are rare and no field studies have been performed to determine the exact sequence of events that lead to infection of bee stings and if not handled properly can lead to fatal outcomes. Our patient responded well to antibiotics and corticosteroids and is presently symptomatically free. Surgical drainage was not required; we report a case of bilateral empyema as a complication of mediastinitis associated with bee sting.

## References

[1] Harry R, Riches C (1982). Hypersensitivity to Bee venom. *Bee World*.

[2] Lazoglu AH, Boglioli LR, Taff ML, Rosenbluth M, Macris NT (1995). Serum sickness reaction following multiple insect stings. *Annals of Allergy, Asthma and Immunology*.

[3] Li JTC, Yunginger JW (1992). Management of insect sting hypersensitivity. *Mayo Clinic Proceedings*.

[4] Reisman RE (1991). Unusual reactions to insect venoms. *Allergy Proceedings*.

